# Vitellogenins - Yolk Gene Function and Regulation in *Caenorhabditis elegans*

**DOI:** 10.3389/fphys.2019.01067

**Published:** 2019-08-21

**Authors:** Marcos Francisco Perez, Ben Lehner

**Affiliations:** ^1^Centre for Genomic Regulation (CRG), The Barcelona Institute of Science and Technology, Barcelona, Spain; ^2^Universitat Pompeu Fabra (UPF), Barcelona, Spain; ^3^Institució Catalana de Recerca i Estudis Avançats (ICREA), Barcelona, Spain

**Keywords:** vitellogenin, yolk, embryogenesis, regulation, roundworm, elegans, gene, nematode

## Abstract

Vitellogenins are a family of yolk proteins that are by far the most abundant among oviparous animals. In the model nematode *Caenorhabditis elegans*, the 6 vitellogenins are among the most highly expressed genes in the adult hermaphrodite intestine, which produces copious yolk to provision eggs. In this article we review what is known about the vitellogenin genes and proteins in *C. elegans*, in comparison with vitellogenins in other taxa. We argue that the primary purpose of abundant vitellogenesis in *C. elegans* is to support post-embryonic development and fertility, rather than embryogenesis, especially in harsh environments. Increasing vitellogenin provisioning underlies several post-embryonic phenotypic alterations associated with advancing maternal age, demonstrating that vitellogenins can act as an intergenerational signal mediating the influence of parental physiology on progeny. We also review what is known about vitellogenin regulation – how tissue-, sex- and stage-specificity of expression is achieved, how vitellogenins are regulated by major signaling pathways, how vitellogenin expression is affected by extra-intestinal tissues and how environmental experience affects vitellogenesis. Lastly, we speculate whether *C. elegans* vitellogenins may play other roles in worm physiology.

## Introduction: Vitellogenins Across the Tree of Life

Vitellogenins are the principal yolk proteins by which oviparous animals supply nutrients to support the development of their progeny. Vitellogenins are large lipo-glyco-phosphoprotein that are expressed in somatic tissues, such as the vertebrate liver (Romano et al., 2004) or the insect fat body (Tufail et al., 2014). They recruit lipids and other nutrients before they are secreted into circulation (e.g., vertebrate blood or insect hemolymph) and are taken up by oocytes via receptor-mediated endocytosis. In vertebrates, vitellogenins are additionally cleaved after uptake within yolk platelets by cathepsins to form lipovitellins and phosvitins. Lipovitellins are larger hydrophobic subunits that carry lipids, while phosvitins are smaller subunits with a high degree of phosphorylation (Raikhel and Dhadialla, 1992).

Vitellogenins form a gene superfamily found in nearly all oviparous taxa (Sappington and Raikhel, 1998), including the monotreme mammals (Brawand et al., 2008). Vitellogenins display a high degree of structural conservation, though they are divergent in regulation and copy number (Tufail et al., 2014). Vitellogenins are believed to be the ancestor of human apoB, the principal component of low density lipoprotein (LDL), on the basis of sequence similarity (Baker, 1988).

While vitellogenin regulation is diverse, they are usually regulated in a sex-, tissue- and stage-specific manner, being expressed in specific somatic tissues of adult females (Raikhel and Dhadialla, 1992). However, vitellogenins are often expressed in male animals to some extent, e.g., in the male honeybee *Apis mellifera* (Piulachs et al., 2003). Vitellogenins are subject to hormonal regulation at the transcriptional level in insects and vertebrates (Tufail et al., 2014). In vertebrates vitellogenins are under estrogenic control. Expression in male animals can be induced by exogenous application of 17-β estradiol (Skipper and Hamilton, 1977).

A wealth of studies indicate that vitellogenins may have additional roles in organismal physiology beyond supporting embryonic development, including caste determination in *A. mellifera* (Guidugli et al., 2005; Nelson et al., 2007), immune functions (Shi et al., 2006; Li et al., 2009; Liu et al., 2009; Tong et al., 2010; Salmela et al., 2015; Sun and Zhang, 2015), antioxidant activity (Seehuus et al., 2006) and metal ion transport (Mitchell and Carlisle, 1991; Falchuk, 1998; Amdam et al., 2004).

## Vitellogenins in *C. elegans* Physiology

*Caenorhabditis elegans* vitellogenins are synthesized in the intestine of the adult hermaphrodite and transported into the germline (Kimble and Sharrock, 1983). The process of activating abundant yolk production at sexual maturation is often referred to as vitellogenesis. Yolk complexes are secreted into the pseudocoelom (body cavity), from where they pass through the gonadal basal lamina and through the 500 nm sheath pores of the somatic gonad (Hall et al., 1999) before uptake by maturing oocytes via receptor-mediated endocytosis (Grant and Hirsh, 1999).

While an appreciation of the characteristics and scale of *C. elegans* vitellogenesis is important for understanding the physiology of this species, the yolk proteins of *C. elegans* also serve as an illustrative and well-characterized lens through which we can observe how the integration of metabolic and environmental pressures and signals affect animal energy homeostasis, aging and even the physiology of the next generation. To that end, in this section “Vitellogenins in *C. elegans* physiology,” we will first explore how the broadly conserved characteristics of vitellogenins and the massive scale of their synthesis affects the physiology of adult worms and their young. In section “Regulation of Vitellogenins in *C. elegans*,” we will go on to detail the molecular mechanisms by which the intestinal production of yolk is tuned, including in response to signals from other tissues and to environmental experience. Finally, in section “Possible Alternative Functions of Vitellogenins in *C. elegans*,” we will speculate on whether the various interesting alternative physiological roles of vitellogenins described in section “Introduction: Vitellogenins Across the Tree of Life,” might also exist in *C. elegans*.

### Molecular Characteristics of *C. elegans* Vitellogenin

Four yolk polypeptides are found in *C. elegans* - two larger polypeptides with a molecular weight of around 170 kDa (YP170A and YP170B) and two smaller polypeptides at around 115 kDa and 88 kDa (YP115 and YP88, respectively). These polypeptides associate to form 2 distinct large oligomeric lipoprotein complexes. The B complex, with a molecular weight estimated at 437,000 kDa, contains only YP170B as a simple dimer, as is typical of vitellogenins in other species. The A complex, with an estimated weight of 439,000 kDa, is an oligomer composed of YP170A, YP115 and YP88. The diameter of these yolk complexes is estimated at 12.8–14.6 nm (Sharrock et al., 1990).

Vitellogenins have been found in multiple proteomic studies to be associated with lipid droplets, likely recruiting lipids during yolk biogenesis (Zhang et al., 2012; Vrablik et al., 2015). Yolk complexes purified from embryos contain around 15% lipid by weight (Sharrock et al., 1990), although this may be an underestimate of the total lipid content of yolk prior to endocytosis, as some lipids likely dissociate from yolk complexes once imported into oocytes. The phospholipids phosphatidylcholine and phosphatidylethanolamine comprise over half of the total lipid content. Neutral lipids constitute around 30%, with free fatty acids and small amounts of cholesterol (Matyash et al., 2001) making up the remainder (Kubagawa et al., 2006; Table 1).

**TABLE 1:** Composition of lipid fraction of yolk isolated from hermaphrodite body cavity.

**Lipid distribution (percentage)**	
Phosphatidylcholine	23.0
Phosphoethanolamine	28.2
Other phospholipids	2.8
Diacylglycerides	3.2
Free fatty acids	16.2
Triacylglycerides	26.4
Cholesterol	<1.0

*Adapted from Kubagawa et al. (2006).*

For *C. elegans* cholesterol is essential but is only required in small quantities. The maternal supply of cholesterol via yolk is sufficient to support normal growth and development under laboratory conditions, with widespread defects in growth and development failing to become apparent until the third generation grown under conditions of cholesterol deprivation (Yochem et al., 1999).

### Vitellogenin Genes in *C. elegans*

**FIGURE 1:**
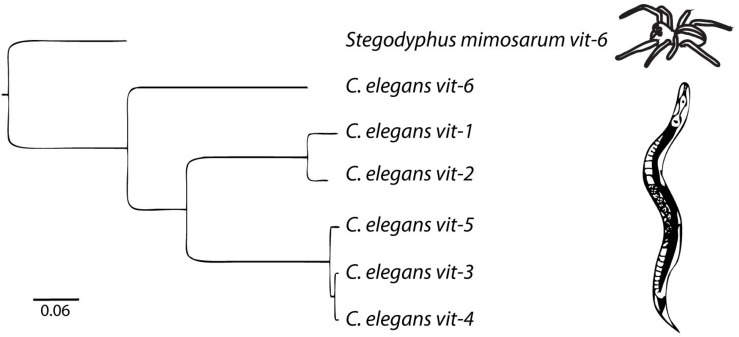
The vitellogenin family in *C. elegans* is comprised of 6 genes. The outgroup is the *vit-6* gene from the African social velvet spider, *Stegodyphus mimosarum*. Arachnid vitellogenins are the sister group to nematode vitellogenins (Tufail et al., 2014). Scale bar represents nucleotide substitutions per site. Adapted from Perez (2018).

There are six vitellogenin genes in the *C. elegans* genome (Figure 1). *vit-2*, the best-studied of the vitellogenins (Goszczynski et al., 2016), encodes YP170B. *vit-1* is 82% identical to *vit-2*. *vit-1* was thought to be a pseudogene based on a report of a single nucleotide deletion causing a truncated protein product (Spieth et al., 1985) It was also noted that while the other vitellogenins are 85–90% conserved between *C. elegans* and the closely related species *Caenorhabditis briggsae*, *vit-1* is only 73% conserved (Blumenthal et al., 1984). However, the reported deletion mutation in the *vit-1* coding sequence is absent in the latest *C. elegans* reference genome (WBcel235) and the VIT-1 protein has been reported to be found in multiple proteomics studies (Zhang et al., 2012; Depuydt et al., 2013; Liang et al., 2014), suggesting that *vit-1* is indeed transcribed and translated.

*vit-3*, *vit-4*, and *vit-5* encode YP170A. *vit-3* and *vit-4* are 99% identical and are arranged in tandem on the X chromosome. *vit-3* and *vit-4* are derived from a recent duplication event, as *C. briggsae* lacks *vit-4* (Blumenthal et al., 1984). *vit-5* is 96% identical to *vit-3* and *vit-4*, and 67% identical to *vit-2* (Spieth et al., 1985).

*vit-6* is the single divergent member of the *C. elegans* vitellogenins, sharing only 50% identity with *vit-2* (Spieth et al., 1985). *vit-6* encodes a single polypeptide of around 180 kDa that is cleaved after secretion into the pseudocoelom but before endocytosis into the oocyte to form YP115, derived from the C-terminal portion of VIT-6, and YP88, derived from the N-terminal portion (Sharrock, 1984; Spieth and Blumenthal, 1985). Despite being cleaved into two separate polypeptides, YP115 and YP88 appear to behave as a single monomeric unit and are linked covalently to YP170A via disulfide bonds (Sharrock et al., 1990). *vit-6* is the only *C. elegans* vitellogenin that is not on the X chromosome, being found instead on chromosome IV.

Although nematode vitellogenins are homologous with vertebrate and other vitellogenins (Spieth et al., 1985; Nardelli et al., 1987), all nematode vitellogenins lack the serine-rich phosvitin domain common to vitellogenins of other taxa (Nardelli et al., 1987; Spieth et al., 1991). Although it is considerably diverged, *vit-6* is much more closely related to the other nematode vitellogenins than to vitellogenins from other taxa, suggesting that this divergence likely occurred within nematodes (Spieth et al., 1991).

*vit-1*, *vit-2*, *vit-3*, *vit-4*, and *vit-5* all have exceptionally short 5′ UTRs (Spieth et al., 1985), ranging from 9–11 bp. This may be related to the presence of potential stem-loop forming structures at the 5′ end of all the vitellogenins, highly conserved between *C. elegans* and *C. briggsae*, that could serve to impede translation (Zucker-Aprison and Blumenthal, 1989), potentially mediating post-translational regulation of vitellogenin expression. All of the vitellogenins lack spliced leader sequences and thus are unlikely to be *trans*-spliced (Sornda et al., 2019).

Although *vit-6* shares only around 21% identity at the protein level with other vitellogenins, all of their amino acid compositions are strikingly similar (Spieth et al., 1991), perhaps reflecting their role in providing offspring with an optimal pool of amino acids. In all vitellogenins there has also been strong conservation of cysteine residues, particularly present in pairs near both termini of the genes (Spieth et al., 1991). These residues are likely required for the formation of disulfide bonds. The *vit* genes also show evidence of strong selection at the level of codon choice (Spieth et al., 1991). Such a strong preference for certain codons is typical of other abundantly expressed nematode genes, such as collagen, actin or myosin.

### Secretion and Uptake of Vitellogenins

**FIGURE 2:**
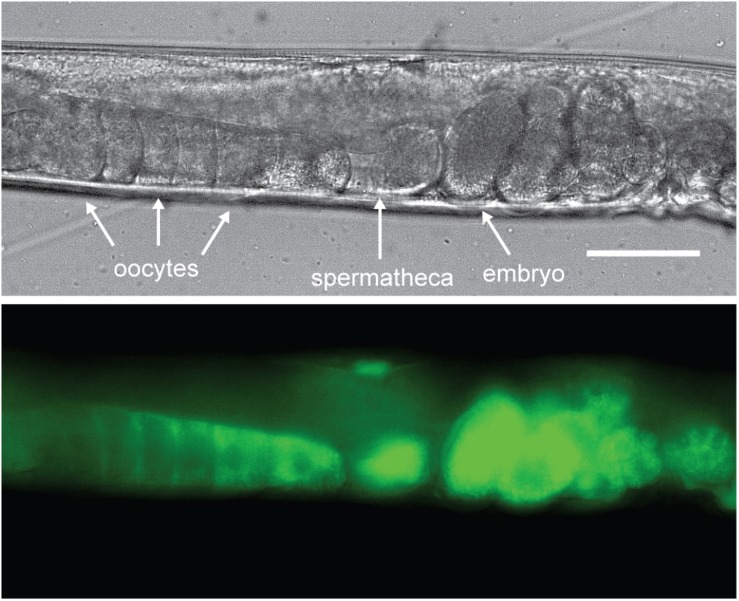
Vitellogenins are taken up by receptor-mediated endocytosis. *C. elegans* oocytes pass through the spermatheca for fertilization, after which the embryos begin development in the maternal uterus. Here a VIT-2:GFP translational fusion protein is shown being taken up by proximal oocytes prior to fertilization. As the oocyte nears the spermatheca, VIT-2:GFP taken up by receptor-mediated endocytosis at the cell membrane gradually extends throughout the cytoplasm and can be seen in the embryos *in utero* after fertilization. Scale bar, 50 μm. Reprinted from Perez (2018).

Yolk in *C. elegans* is loaded into the 3 most proximal oocytes by receptor-mediated endocytosis (Figure 2; Grant and Hirsh, 1999; Hall et al., 1999). The *C. elegans* yolk receptor is encoded by *rme-2*, a member of the low-density lipoprotein receptor (LDLR) superfamily. Embryos of *rme-2* null mutants contain no detectable yolk (Grant and Hirsh, 1999). After endocytosis, yolk appears in oocytes and embryos in a membrane-bound compartment, often referred to as yolk granules (Hall et al., 1999). Various non-protein components taken up with yolk, such as the fluorescent fatty-acid analog BODIPY-FA (Kubagawa et al., 2006), the cholesterol analog dehydroergosterol (Matyash et al., 2001) and fluorescently labeled dsRNA (Marré et al., 2016), have been found to undergo a rapid re-localization away from the yolk granules to the cytoplasm or other cellular organelles. This implies that yolk is actively sorted into various components shortly after uptake.

The process of vitellogenin secretion and uptake is very fast, with labeled yolk particles undergoing transport from the intestine to the germline within minutes (Bossinger and Schierenberg, 2003; Kuo et al., 2013).

In the course of embryogenesis, yolk accumulates in the gut primordium (Sharrock, 1983). Yolk deposition in the gut by various mechanisms, such as prelocalization or segregation, is common in other taxa (Bossinger and Schierenberg, 2003). In the case of *C. elegans*, yolk appears to be secreted from other cells and taken up again by the gut cells by receptor-mediated endocytosis (Bossinger and Schierenberg, 2003; Kuo et al., 2013). As *rme-2* is not expressed during embryogenesis (Grant and Hirsh, 1999), this implies the existence of another receptor capable of mediating yolk uptake, possibly in a processed form, in the worm.

### *C. elegans* Vitellogenins Are Highly Expressed

The gonad comprises around one quarter of an adult hermaphrodite’s body volume and turns over every 6.5 h at the peak of the reproductive period (Hirsh et al., 1976; Jung et al., 2012). Therefore, in 1 day the worm produces a quantity of embryos roughly equivalent to its own body weight. It has been estimated that yolk comprises 37% of the total protein in the embryo (Kimble and Sharrock, 1983). Kimble and Sharrock (1983) found that YP170 alone comprised 25% of *de novo* protein synthesis from dissected young adult hermaphrodite intestines *in vitro*. Van Nostrand et al. (2013) found that the genes encoding vitellogenins are the protein-coding genes with the highest expression in young adult worms, constituting around 3% of all RNA-seq reads. This is despite the fact that they are expressed exclusively in the 20 cells of the intestine and despite the fact that yolk synthesis increases further as worms get older (Perez et al., 2017; Sornda et al., 2019).

With these figures in mind, we might be justified in proposing that a primary purpose of the adult hermaphrodite worm is to produce prodigious quantities of yolk to provision her embryos, at a presumably huge metabolic cost. As such, in a healthy adult hermaphrodite the expression of vitellogenins may be near saturation and limited primarily by the biosynthetic capacity of the intestine. This hypothesis is supported by the observation that almost all other genes expressed in the intestine increased their expression upon knockdown of *unc-62*, a necessary transcriptional activator of vitellogenins (Van Nostrand et al., 2013). Similarly, knockdown of all vitellogenin genes by RNAi increased the abundance of numerous other proteins in aged worms (Sornda et al., 2019). The massive scale of yolk synthesis may be the reason for the existence of multiple, near-identical vitellogenin paralogs in the *C. elegans* genome and also for the endoreduplication of DNA that occurs in intestinal cells, reaching completion shortly before the onset of reproduction (Hedgecock and White, 1985). Most adult hermaphrodite intestinal cells contain 64 haploid genomes, equating to almost 400 copies of the *vit* genes per cell. Overexpression of these genes as a group may therefore be difficult to attain experimentally. Adding additional gene copies may simply bias the composition of the total yolk pool toward one or another isoform according to the identity of the added gene, as appears to be the case at the level of transcription (Spieth et al., 1988) and translation (Van Rompay et al., 2015; Sornda et al., 2019). Claimed overexpression of vitellogenins therefore requires data on all vitellogenins, ideally at the protein level, and not just the expression level of the added gene. The increase in yolk synthesis that occurs during the reproductive period may be supported by a concurrent increase in the volume of the hermaphrodite intestine (Perez et al., 2017).

**FIGURE 3:**
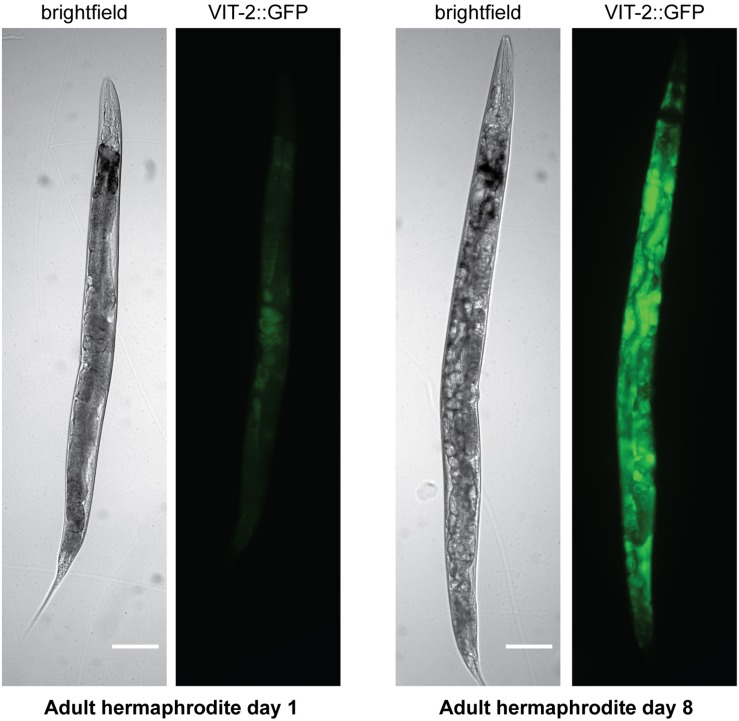
Yolk accumulates to pathological levels in post-reproductive hermaphrodites. Brightfield and fluorescence images are shown of hermaphrodites on adult day 1 or adult day 8 carrying a VIT-2:GFP translational fusion. Scale bar, 50 μm. Reprinted from Perez (2018).

Yolk also accumulates in the adult hermaphrodite toward the end of the self-fertile reproductive period (Perez et al., 2017; Sornda et al., 2019), such that elevated embryonic yolk uptake may depend more on the level of accumulated yolk rather than the rate of *de novo* synthesis at this point. In aged worms, yolk accumulates to pathological levels (Herndon et al., 2002; Figure 3). The accumulation of yolk has been understood as at least partly a consequence of the cessation of reproduction, causing the absence of the main yolk sink by which the massive quantities of yolk produced leave the body (McGee et al., 2011; Sornda et al., 2019). However, although yolk production does decline from its peak (Van Nostrand et al., 2013), aging worms do continue to synthesize some yolk *de novo*, even up to day 10 of adulthood or later (Liang et al., 2014; Sornda et al., 2019) as a result of the inappropriate continuation of the programed massive yolk synthesis during the reproductive period (Ackerman and Gems, 2012; Gems and de la Guardia, 2013). *vit* RNAi extends lifespan (Murphy et al., 2003; Seah et al., 2016), and it had been suggested that it does so by preventing lipotoxicity associated with the accumulation of yolk during aging. However, it is unclear whether knockdown of *rme-2*, which also leads to build-up of yolk in extracellular spaces, reproducibly influences lifespan (Seah et al., 2016; Dowen, 2019), casting doubt on the significance of yolk toxicity in aging. This apparent contradiction appears to be resolved by a study which suggests that it is not toxic yolk accumulation which causes aging-related pathologies but rather the autophagy-dependent degradation of the worm’s intestinal tissues to fuel the continued massive production of yolk (Ezcurra et al., 2018).

### Vitellogenins Are Dispensable for Embryonic Development in *C. elegans*

Although it has been widely assumed in the literature that abundant vitellogenin expression is required for adult fertility and embryonic viability, a body of evidence suggests this is not the case. Vitellogenin knockdown in adulthood is reported to have little impact on fecundity (Ezcurra et al., 2018). Likewise, multiple mutants that almost completely abrogate synthesis of all vitellogenins in the adult hermaphrodite have little or no effect on brood size, reproductive timing or embryo viability (Van Rompay et al., 2015). Viability is severely affected in embryos of a *rme-2* null mutant, *rme-2*(*b1008*), which appear completely devoid of any detectable yolk, but 23% are still viable (Grant and Hirsh, 1999), demonstrating that yolk is not strictly required for embryogenesis. *rme-2*(*b1008*) hermaphrodites do have a severely reduced brood size (78 compared to ∼300 in N2) (Grant and Hirsh, 1999), but the full brood size of almost-yolkless mutants indicates that this is due to defects in ovulation and fertilization. Many *rme-2* oocytes are destroyed during the passage through the spermatheca (Grant and Hirsh, 1999). Chi and Reinke (2009) demonstrated that *rme-2* was required upstream of inositol triphosphate (IP3) signaling to control proper dilation of the spermathecal valve. The low surviving brood size phenotype of *rme-2*(*b1008*) mutants was dramatically suppressed by introducing mutations in IP3 signaling. This shows that many *rme-2* mutant embryos die due to damage to the oocyte during fertilization, rather than as a result of a lack of yolk.

Additionally, oocytes require polyunsaturated fatty acids (PUFAs) supplied by yolk in order to produce hormonal signals, likely prostaglandins (Edmonds et al., 2010), to attract sperm and complete successful fertilization (Jacinto et al., 2004; Kubagawa et al., 2006). The migration of sperm toward oocytes is also necessary for sperm to regain their position after being pushed into the uterus by oocytes passing through the spermatheca. Hermaphrodites with sperm guidance defects often exhibit a reduced brood size due to consequent loss of self-sperm through the vulva (Han et al., 2009), which may also contribute to the low brood size of *rme-2* null mutants. We speculate that in mutants that almost abrogate yolk synthesis (Van Rompay et al., 2015), the small amount of PUFAs delivered by the little remaining yolk suffices to produce the necessary signal to attract sperm to the oocyte and thus allow approximately normal brood sizes.

### Vitellogenins Support Post-embryonic Development and Can Mediate Intergenerational Effects of Parental Physiology

**FIGURE 4:**
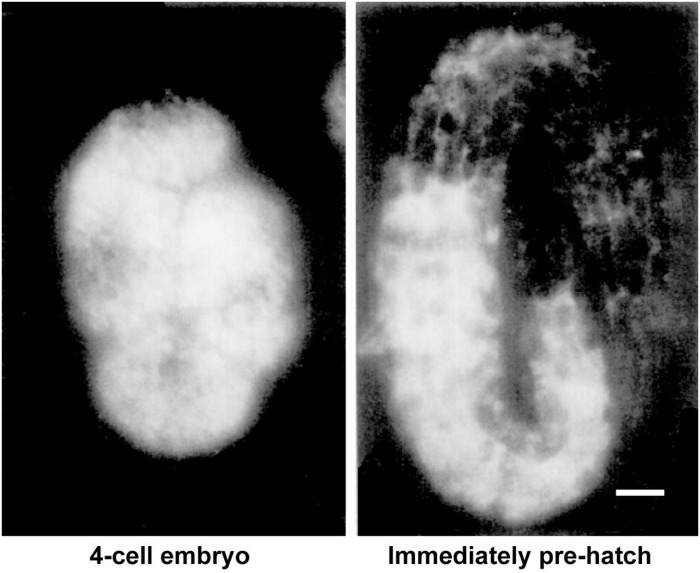
Substantial levels of vitellogenin remain after embryogenesis. Vitellogenin visualized by immunofluorescence in an early embryo and in an embryo immediately prior to hatching. Scale bar, 5 μm. Adapted from Sharrock (1983).

If yolk is largely dispensable for embryogenesis, why does the worm expend so much energy producing it in staggering quantities? The answer seems to be that abundant yolk supports post-embryonic progeny survival and development. This was originally suggested on the basis of the significant proportion of yolk left in the larvae after embryogenesis has been completed (Figure 4; Sharrock, 1983; Bossinger and Schierenberg, 2003). Indeed, mutants defective in yolk synthesis (Van Rompay et al., 2015), yolk endocytosis or appropriate yolk utilization (Chotard et al., 2010) have impaired survival of larval starvation.

**FIGURE 5:**
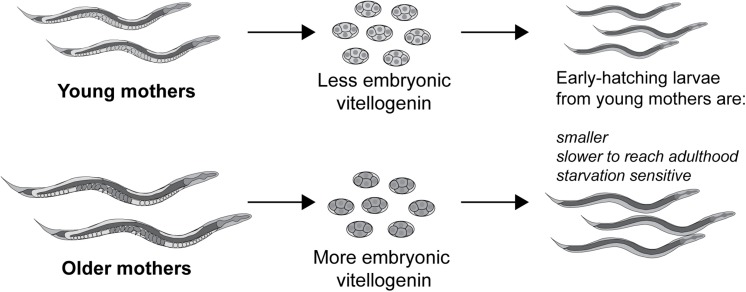
Vitellogenin underlies the small size and susceptibility to L1 starvation of the progeny of young mothers. Vitellogenin provisioning to embryos increases during the self-fertile reproductive period, possibly due to an increase in biosynthetic capacity in older, larger worms. As a result of their higher embryonic vitellogenin titer, progeny of older mothers are larger at hatching, are more resistant to L1 starvation and reach adulthood sooner, even if the absence of starvation. Conversely the early progeny coming from younger mothers, with the least embryonic vitellogenin, are impaired for these same traits.

**FIGURE 6:**
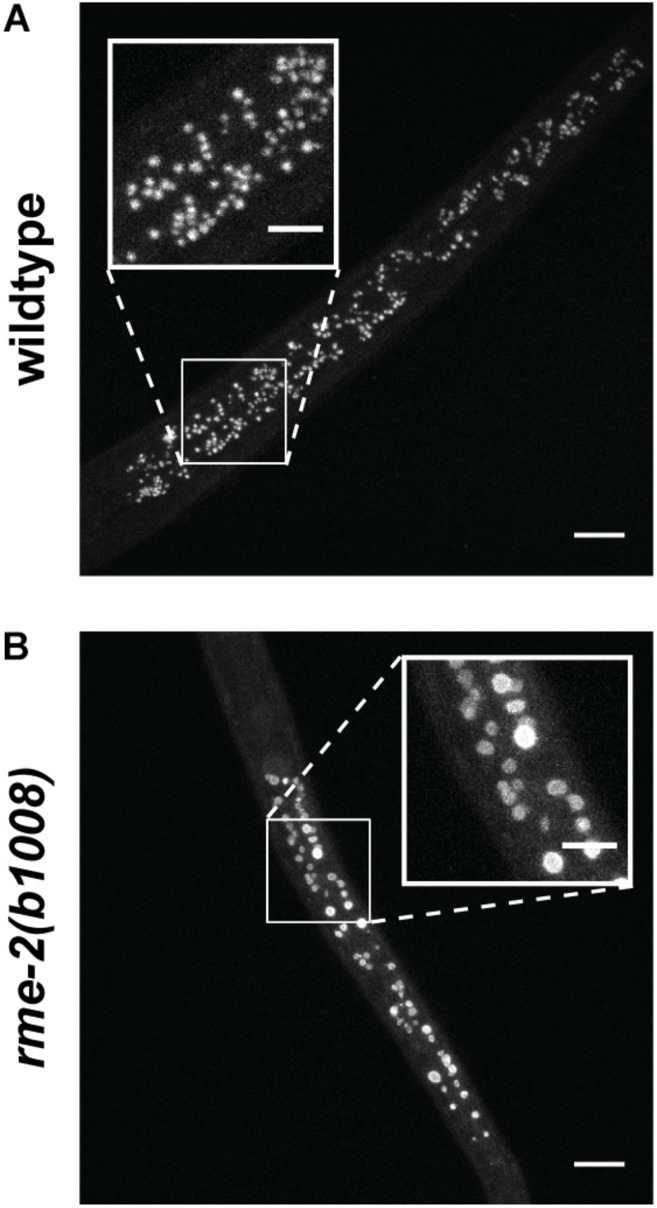
Autofluorescent gut granules are grossly enlarged upon hatching in larvae mutant for the single yolk receptor, *rme-2*. **(A)** Blue autofluorescence from lysosome-related organelles in newly hatched wildtype L1 larva. **(B)** Newly hatched L1 larvae of *rme-2*(*b1008*) mutants, which lack any detectable embryonic yolk, have very large lysosome-related organelles. This may be due to the abrogation of phospholipid import by vitellogenin lipoprotein complexes. The physiological consequences of this enlargement are unknown. Scale bars main images 10 μm, insets 5 μm. Reprinted from Perez (2018).

It was shown that embryonic vitellogenin titers increase with maternal age during the reproductive period (Figure 5; Perez et al., 2017). Low vitellogenin provisioning to the progeny of young mothers leads to shorter length at hatching and larger autofluorescent lysosome-related organelles, commonly known as gut granules, with unknown physiological consequences (Perez et al., 2017). This gut granule phenotype is grossly exaggerated in larvae of *rme-2* mutants (Figure 6; Perez, 2018) and may result from a constraint on the surface area-to-volume ratio of membrane-bound organelles due to limited maternal supply of phospholipids, the major component of the lipids transported by vitellogenin lipoprotein complexes (see Table 1). In addition to larger size at hatching, the increased vitellogenin provisioning in progeny of older mothers underlies a corresponding resistance to L1 starvation. Worms that experience a long L1 starvation often show defects in post-embryonic development. These defects are most commonly germline abnormalities, often leading to complete sterility, but also include somatic defects such as multivulva, protruding vulva or bursting. Worms hatched from vitellogenin-depleted embryos, achieved by maternal knockdown of either vitellogenins or *rme-2*, show an increased frequency of these defects, including sterility, as do the progeny of young mothers (Perez et al., 2017; Jordan et al., 2019). Accordingly, progeny of young mothers, with reduced vitellogenin titers, showed premature signs of aging (Roux et al., 2016) during L1 starvation (Olmedo et al., 2018). Increased embryonic vitellogenin titers lead to reduced insulin-like signaling in progeny, which mediates the supression of germline defects after recovery from L1 starvation (Jordan et al., 2019) and may also explain the supression of aging in starved L1s. Additionally, increased vitellogenin in the embryos of older mothers explains a shortening of the time taken to reach adulthood after hatching, even in the absence of starvation (Perez et al., 2017). It was shown that young maternal age and embryonic vitellogenin depletion by *rme-2* RNAi led to delayed adulthood after feeding in previously-starved L1s not by accelerating the overall rate of development but specifically prolonging the time required to initiate development from the L1 stage (Olmedo et al., 2018). High levels of yolk provisioning thus improve offspring outcomes, in both favorable and unfavorable conditions.

The “boom-and-bust” mode of rapid population growth on ephemeral, dispersed resource patches (Félix and Braendle, 2010) means few individuals will survive to proliferate outside their “home” patch. Even when food is plentiful, competition will be intense and a short generation time is crucial. Yolk supports progeny survival in the absence of food and reduces the time needed until a fed larva can produce her own eggs. The scale of yolk production in *C. elegans* indicates that the worm places a premium on the ability of its progeny to survive harsh conditions and thrive in benign environments.

The example of maternal age illustrates that alteration of vitellogenin provisioning also acts as an intergenerational signal through which parental physiology can influence progeny outcomes throughout development and into adulthood. Since August Weismann proposed the concept of the separation of the soma and the germline, biologists have tended to consider the germline, and consequently the phenotypic traits of an individual’s descendants, as free from the influence of the somatic tissues and thus much physiological and environmental influence. In recent decades this view has been challenged, with a focus on so-called epigenetic mechanisms of inheritance, such as DNA methylation, chromatin modifications or inheritance of small RNA populations, although the details of how these inherited factors are influenced by somatic tissues remain elusive (Perez and Lehner, 2019). It has thus been overlooked that the process of vitellogenesis and oocyte provisioning in almost all oviparous species represents a massive and direct flow of matter from the soma into the germline, with the obvious potential to alter progeny phenotypes as demonstrated in *C. elegans*. Moreover, the huge metabolic cost of vitellogenesis in the worm, and presumably many other species, dictates that yolk production in hermaphrodites be tightly controlled by a variety of conserved metabolic pathways which are known to mediate the physiological response of *C. elegans* to its environment, as covered in section “Regulation of Vitellogenins in *C. elegans*,” of this review. Vitellogenins thereby represent a likely intergenerational mechanism by which the environment of *C. elegans* parents can act to influence progeny phenotypes.

## Regulation of Vitellogenins in *C. elegans*

**FIGURE 7:**
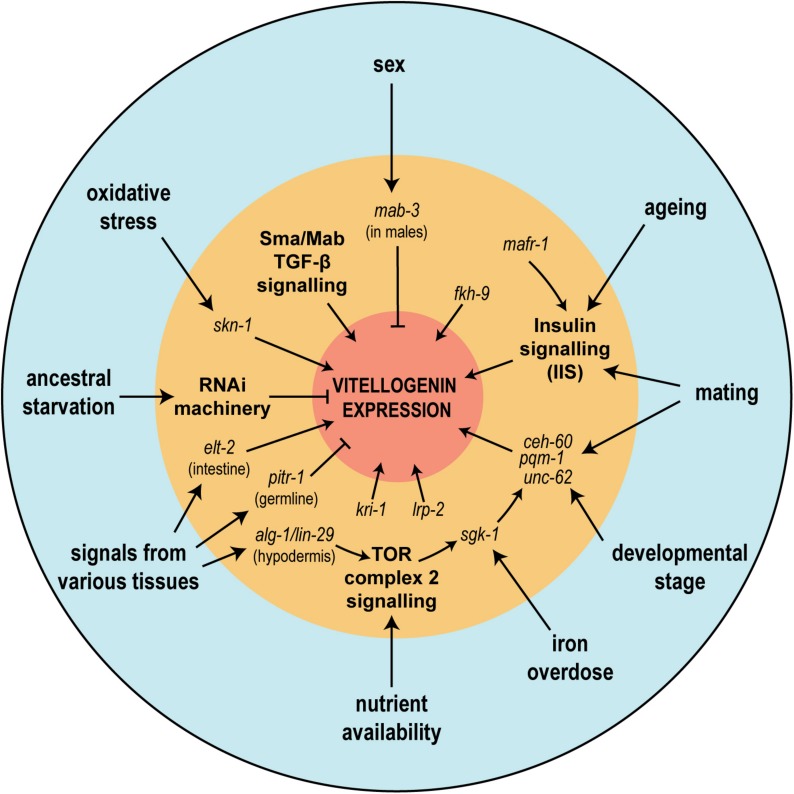
The regulation of vitellogenin genes is affected by various physiological and environmental conditions upstream of a multitude of signaling pathways. In the figure, physiological or environmental factors known to influence vitellogenin expression are seen in the outermost blue circle. In the intermediate orange circle are shown the various described genetic regulators or signaling pathways (bold), many of which have been shown to mediate physiological or environmental effects. Together these regulators tightly control the abundant and metabolically costly expression of the vitellogenin genes (represented by the central red circle).

The vitellogenins are subject to tight regulatory constraints; their abundant expression needs to be confined in a tissue-, stage- and sex- specific manner, limited to the intestine of the adult hermaphrodite worm. As the metabolic cost of abundant vitellogenin expression must be staggering, fine-tuning of their expression is paramount; hence multiple regulatory inputs exist, integrating signals stemming from nutritional status, environmental conditions and extra-intestinal tissues (Figure 7). It has often been stated in the literature that the promoters of the *vit* genes are simple (e.g., MacMorris et al., 1992), but recent extensive genetic dissection has found multiple regulators and signaling pathways impinging directly and indirectly on only a small section of the promoter of *vit-2* (Goszczynski et al., 2016), the most extensively studied of the vitellogenins.

Many studies have claimed to find differential regulation of vitellogenins, but in the light of the recent findings by ourselves and others that vitellogenin expression throughout adulthood is highly dynamic (Perez et al., 2017; Ezcurra et al., 2018), caution is required as the likelihood of artifacts is high in any genetic or environmental context that alters developmental timing. With noteworthy exceptions (e.g., Van Rompay et al., 2015; Goszczynski et al., 2016) few studies have explicitly controlled for this.

Of note, it is important to distinguish carefully between vitellogenin expression in adults and vitellogenin titers in embryos when discussing the impacts of signaling pathways or genetic regulators. It is possible for a perturbation to influence expression and provisioning in seemingly contradictory ways, if the perturbation also changes the rate of embryo production at a given point in time (Perez, 2018). For example, this appears to be the case for dietary restriction, which has been reported to suppress vitellogenin expression in adults (Seah et al., 2016) but increase vitellogenin titers in embryos (Jordan et al., 2019), likely due to a concurrent reduction in the production of embryos (Hibshman et al., 2016). In these sections, we refer to the regulation of vitellogenin expression in adults unlesss specifically noted otherwise.

First we will look at the basic logic of the regulation of vitellogenins, which determines the localization and timing of their expression. To continue, we will look at the major signaling pathways that have been identified as regulating vitellogenins. We will also briefly touch on how other tissues, through these pathways or others, can exert influence on vitellogenin expression in the intestine. Lastly, we mention a few of the environmental influences that have been shown to influence vitellogenin expression.

### Tissue-, Stage- and Sex-Specific Regulation

The primary regulatory logic of high yolk expression in a tissue-, stage- and sex-specific manner can be recapitulated with only 247 bp of the *vit-2* promoter (MacMorris et al., 1992). Even a minimal 44 bp enhancer region from the *vit-2* promoter 145–188 bp upstream of the initiation codon allows for full recapitulation of sex- and tissue-specificity and partial recapitulation of stage-specificity, with only weak expression evident in developing larvae (Goszczynski et al., 2016).

Three functionally associated sequence elements are found in the vitellogenin promoters. Two have been christened *vit* promoter elements, VPE1 (TGTCAAT) and VPE2 (CTGATAA; MacMorris et al., 1992), while the third is a direct binding site for the transcription factor MAB-3 [AATGTTGCGA(T/A)NT; Shen and Hodgkin, 1988; Yi and Zarkower, 1999]. Remarkably there is considerable conservation of these promoter elements across vast phylogenetic distances - the exact VPE2 sequence is found upstream of vitellogenin genes in *Xenopus laevis* and *Gallus gallus*, with very similar sequences also found in *Drosophila melanogaster* (Spieth et al., 1985).

VPE2 confers abundant expression in a tissue-specific manner. VPE2 is directly bound by the intestinal master regulator ELT-2 (McGhee et al., 2007). Knockdown of *elt-2* in young adult worms strongly reduces expression from a 44 bp enhancer region of the *vit-2* promoter (Goszczynski et al., 2016).

VPE1 is bound by an isoform of the transcription factor UNC-62, which is necessary for vitellogenin expression. Isoform-specific RNAi targeting *unc-62a* reduces vitellogenin expression between four- and 10-fold (Van Nostrand et al., 2013). As *unc-62a* undergoes a dramatic increase in intestine-specific expression between the L3 stage and adulthood (Van Nostrand et al., 2013) it likely confers some stage-specificity, although Goszczynski et al. (2016) found that a 44 bp promoter element that does not contain VPE1 still recapitulated stage-specificity to some extent. UNC-62 has been proposed to regulate vitellogenesis as part of a multimeric complex with another transcription factor, CEH-60 (see section “Other Regulators”), and possibly PQM-1 (see section Regulation by SKN-1; Dowen, 2019).

Sex-specificity is conferred by the MAB-3 binding site, as *mab-3* mutant males accumulate yolk (Yi and Zarkower, 1999). *mab-3* is homologous to the *Doublesex* gene of *D. melanogaster*, which also represses yolk protein transcription (Yi and Zarkower, 1999). MAB-3 has been shown experimentally to act directly on the *C. elegans vit-2* promoter, but all vitellogenins contain potential MAB-3 binding sites and all are deregulated in *mab-3* loss of function mutants (Yi and Zarkower, 1999). Direct MAB-3 binding may inhibit activation by *elt-2* or *unc-62* (Goszczynski et al., 2016). *mab-3* acts in the sex determination pathway downstream of *tra-1*, which blocks *mab-3* activity in hermaphrodites and thus relieves the repression of the vitellogenins. Curiously, in a *mab-3* mutant background different *tra-1* alleles have different effects on the relative abundances of the yolk proteins, suggesting that *tra-1* also acts independently of *mab-3* to influence synthesis of specific vitellogenins (Shen and Hodgkin, 1988). Surprisingly, Goszczynski et al. (2016) found that removal of the MAB-3 binding site abolished expression driven by a 44 bp enhancer element of the *vit-2* promoter in both sexes, suggesting an additional unknown regulator necessary for expression also binds to this sequence element.

### Regulation by Signaling Pathways

Here we discuss regulation of vitellogenins at the level of individual signaling pathways, although one must bear in mind the extensive and complex interactions that can exist between various pathways. For example, there is extensive cross-talk between the insulin-like signaling and TOR pathways (Narasimhan et al., 2009), with TOR acting both downstream and parallel to insulin-like signaling (Baumeister et al., 2006). Interaction between signaling pathways can be relevant in some tissues or processes but not others (Qi et al., 2017), with various pathways converging on the same downstream targets, such as *sgk-1* and *skn-1* (Robida-Stubbs et al., 2012).

#### Insulin/Insulin-Like Growth Factor Signaling (IIS)

The widely-studied IIS pathway plays a crucial role in the response of *C. elegans* to environmental conditions, such as nutrient availability and various stressors. In turn, IIS regulates pivotal life-history decisions for individual worms, notably L1 and dauer arrest (reviewed in Murphy and Hu, 2013; Baugh, 2013). DAF-2, orthologous to the human insulin receptor, is bound by a plethora of insulin-like peptides, both agonists and antagonists (Murphy, 2006), which lead, via a phosphatidylinositol (3,4,5)-trisphosphate (PIP_3_) signaling cascade, to phosphorylation and consequent cytoplasmic localization of the master stress regulator DAF-16. In the absence of signaling by DAF-2, DAF-16 enters the nucleus and directly or indirectly modulates the expression of thousands of downstream target genes (Murphy, 2006).

The IIS pathway has widely been reported as a repressor of vitellogenins. Multiple studies, particularly of the aging process in *daf-2* mutants, have found that IIS mutants exhibit reduced vitellogenin transcription and accumulation in older worms (Murphy et al., 2003; DePina et al., 2011). This was even suggested to partially mediate the longevity of *daf-2* worms, given that vitellogenin knockdown by RNAi can extend lifespan (Murphy et al., 2003).

DePina et al. (2011) tackled the subject of IIS regulation of vitellogenesis most directly. In worms carrying the pleiotropic class II *daf-2* mutant allele *e1370* (Gems et al., 1998), yolk protein levels were observed to be lower than wildtype, in a *daf-16* dependent manner. However, vitellogenin transcription was similar in *daf-2* mutant and wildtype worms during the self-fertile reproductive period. The authors infer *daf-16*-dependent post-transcriptional regulation of yolk protein production. However, it is unclear in this study whether the *daf-16*-dependent extended reproductive span of *daf-2* mutants (Murphy et al., 2003), which would delay the post-reproductive accumulation of vitellogenins (McGee et al., 2011), may contribute to the apparent difference compared to wildtype worms.

In a thorough dissection of regulatory inputs impinging on a 44 bp “enhancer” element of the *vit-2* promoter, Goszczynski et al. (2016) found that *daf-2* loss of function abolished transcriptional output from the enhancer element in a *daf-16* dependent manner. Furthermore, a *daf-16* null mutant had higher expression driven by the enhancer, suggesting constitutive repression of this element by the IIS pathway. Indeed, they found that both DAF-16 DNA-binding isoforms bound directly to the 44 bp enhancer sequence *in vitro*, although no prior studies had identified vitellogenins as sites of DAF-16 binding *in vivo* using either ChIP-seq (Oh et al., 2006) or DNA adenine methyltransferase identification (DamID; Schuster et al., 2010). *daf-2* was found to exert its influence in a cell non-autonomous manner, as intestine-specific *daf-2* RNAi did not abolish transcription; this is consistent with the reported expression pattern of *daf-2*, which is expressed mainly in neurons (Li et al., 2014) and the germline (Honnen et al., 2012). In contrast, intestinal *daf-16* RNAi partially restores expression in the context of *daf-2* loss of function. This indicates that while there is likely some direct repression of vitellogenins by *daf-16*, some degree of repression comes from *daf-16* activity in other tissues, presumably via cross-talk with other signaling pathways. These results support the notion that IIS is a major regulator of vitellogenesis in *C. elegans*.

However, the same authors also found that despite abolishing expression from the 44 bp enhancer, *daf-2* RNAi had virtually no effect on expression driven by the full 2.7 kb *vit-2* promoter region. The authors conclude that although IIS acts strongly on the 44 bp enhancer, there are likely numerous other signaling pathways converging on the full promoter region, leading to the influence of IIS on vitellogenesis in early adulthood being modest, at most. How to reconcile this with their own results, and the previous consensus of vitellogenins as IIS targets? Characterization of vitellogenins as IIS targets has been largely based on expression studies of aging worms. The notion that IIS plays a major role in the regulation of vitellogenins in the context of post-reproductive yolk accumulation is thus compatible with the idea that it has a minor role, as one of many regulatory players, in vitellogenesis during the reproductive period. Consistent with this idea, a meta-analysis of *daf-16* loss of function expression studies found the regulatory association with vitellogenins to be weak and inconsistent (Tepper et al., 2013).

Embryonic vitellogenin titers are increased in mothers with reduced IIS signaling (Jordan et al., 2019), although the extent of this increase may change with maternal age (Perez, 2018).

#### TGF-β Signaling

The TGF-β superfamily is an extensive class of secreted ligands that play fundamental roles in animal physiology, development and growth. In *C. elegans* two parallel, distinct branches of TGF-β signaling have been characterized - the dauer pathway and the Sma/Mab pathway. Both pathways share the single type II receptor, DAF-4, with specificity of input provided by distinct ligands and type I receptors and specificity of output provided by distinct suites of downstream intracellular signal transducer, the SMADs, and associated transcription factors (Gumienny and Savage-Dunn, 2013). Alongside other signaling pathways, the dauer TGF-β pathway plays a key role in the determination of whether or not to enter the dauer diapause, a decision based on environmental and nutritional status in early development. Loss of signaling results in dauer-constitutive phenotypes.

The Sma/Mab TGF-β signaling pathway regulates post-embryonic growth (Savage-Dunn et al., 2000), germline maintenance and reproductive aging (Luo et al., 2010) and some aspects of male development (Savage et al., 1996). The ligand for this pathway is DBL-1; *dbl-1* loss of function leads to small mutants with reduced body size, while *dbl-1* overexpression results in abnormally long worms (Suzuki et al., 1999). *dbl-1* is expressed principally in neurons (Ramakrishnan et al., 2014). DBL-1 signaling is potentiated by a transmembrane protein SMA-10, which binds to the type I and type II receptors SMA-6 and DAF-4 (Gumienny et al., 2010). SMA-6 phosphorylates the R-SMADs SMA-2 and SMA-3, which associate with the Co-SMAD SMA-4 to influence transcription. No activity of *sma-2*, *sma-3* or *sma-4* can be detected in the absence of any one of the three genes, suggesting that they form a heterotrimeric complex (Savage-Dunn et al., 2000). A subset of Sma/Mab targets require the transcriptional co-factor *sma-9*, encoding a large zinc finger transcription factor homologous to *Schnurri* from *D. melanogaster* (Liang et al., 2003). Transcriptional targets include *lon-1*, which when mutated suppresses the body size phenotype of *sma* mutants (Maduzia et al., 2002). Activity of the pathway in the hypodermis is necessary and sufficient for determining body size phenotypes (Wang et al., 2002).

In an attempt to identify additional transcription factors that interact with the MAB-3 binding site of the 44 bp enhancer element from the *vit-2* promoter, Goszczynski et al. (2016) performed an RNAi screen, targeting 167 of the approximately 200 transcription factors thought to be active in the *C. elegans* intestine. They found a strong repression of transcription resulting from knockdown of the Sma/Mab Co-SMAD *sma-4*, implying that TGF-β signaling promotes vitellogenesis. Indeed, subsequent knockdown of *dbl-1*, *daf-4*, *sma-6*, *sma-10*, *sma-2*, *sma-3*, and *sma-9* all produced a strong repression of transcriptional output from the enhancer element. Intestine-specific RNAi indicated that all of these components acted within the intestine, except for *dbl-1*, consistent with its neuronal expression pattern. Importantly these experiments were conducted with concurrent *lon-1* RNAi, in order to exclude body size as a confounding factor. Similar results were obtained using either 247 bp or 2.7 kb stretches of the *vit-2* promoter. The authors were unable to find a direct association of SMADs or SMA-9 with the 44 bp enhancer element, demonstrating that this regulation is likely to be indirect. The suppression of transcriptional output from the full *vit-2* promoter by knockdown of *sma-9* was ∼40%, suggesting that while important, TGF-β signaling exerts a quantitative influence on vitellogenesis in concert with other signaling pathways.

#### Target-of-Rapamycin (TOR) Signaling

Originally discovered as the protein inhibited by the potent antifungal compound rapamycin, TOR (target of rapamycin) kinase, a serine/threonine kinase of the phosphatidylinositol kinase-related kinase (PIKK) family, is a ubiquitous central regulator of growth in eukaryotes. Every eukaryotic genome examined, bar a handful of fungal pathogens (Shertz et al., 2010), contains a TOR gene (Wullschleger et al., 2006). TOR kinase integrates inputs from growth factors, availability of nutrients (especially amino acids), energy levels and stress conditions, and in turn coordinates transcription and translation, ribosome biogenesis, metabolism and autophagy (Wullschleger et al., 2006).

Target-of-Rapamycin kinase participates in two protein complexes, TORC1 and TORC2, defined in part by TOR’s interaction with two mutually exclusive binding partners, Raptor and Rictor, respectively (Jacinto et al., 2004; Sarbassov et al., 2004). TORC1 controls temporal aspects of growth in yeast and mammalian embryogenesis (Wullschleger et al., 2006); TORC2, in contrast, is known to affect spatial aspects of growth by regulation of the actin cytoskeleton (Sarbassov et al., 2004). In *C. elegans*, TORC2 phosphorylates and activates PDK-1, AKT-1, and AKT-2 (Jones et al., 2009), components of the PIP_3_ signaling cascade linking DAF-2 signaling to DAF-16 activity (Murphy and Hu, 2013), among other targets. However, the principal downstream effector of most of the phenotypes that manifest in *rictor* mutants with compromised TORC2 activity is the serum and glucocorticoid-induced kinase, SGK-1 (Jones et al., 2009; Soukas et al., 2009), which also acts in the IIS pathway downstream of PIP_3_ signaling (Murphy and Hu, 2013). SGK-1 is also capable of phosphorylating DAF-16 *in vitro* (Hertweck et al., 2004).

In a reverse genetic screen for activators of transcriptional output from the *vit-3* promoter at the L4 to adult transition, Dowen et al. (2016) identified mutations in *alg-1*, an Argonaute protein involved in microRNA (miRNA) biogenesis that acts in the hypodermis to promote vitellogenesis (reviewed in section “Hypodermis”). The screen also identified a mutation in *sgk-1* that led to repression of the *vit-3* promoter. *sgk-1* is an excellent candidate for a regulator of vitellogenesis, as it is expressed exclusively in the adult intestine and neurons (Hertweck et al., 2004). It was found that an *sgk-1* gain-of-function mutation could rescue the vitellogenesis defects of mutants in the hypodermal miRNA pathway. As mutations in the PIP_3_ signaling modulator *daf-18* (the *C. elegans* ortholog of human phosphatase and tensin homolog, PTEN) could not rescue expression from the *vit-3* promoter in a hypodermal miRNA pathway mutant, it was determined that this pathway did not act via the IIS pathway. Likewise, the influence of *sgk-1* on the *vit-3* promoter was *daf-16* independent.

Intestinal RNAi against *let-363*, encoding the *C. elegans* homolog of TOR kinase, strongly repressed the *vit-3* promoter. Knockdown of the TORC2 components *rict-1* (*rictor*) and *sinh-1*, but not TORC1-specific components, repressed vitellogenesis, suggesting that TORC2 promotes vitellogenesis through its primary target, SGK-1. Via a forward genetic screen in the *sgk-1* mutant background, it was found that SGK-1 acts through PQM-1, a nematode-specific zinc finger transcription factor (Dowen et al., 2016).

*pqm-1* was previously characterized in a search for the mechanism by which *daf-16* appeared to repress a large class of downstream targets, despite being a direct transcriptional activator. These targets are strongly enriched for intestinal genes and share a DNA motif, dubbed the *daf-16* associated element (DAE) that is bound by PQM-1 (Tepper et al., 2013). Like DAF-16, PQM-1 undergoes a shift in subcellular localization under stress conditions, but curiously exhibits an opposite and mutually antagonistic shift; when DAF-16 is largely cytoplasmic PQM-1 is found in the nucleus, while nuclear DAF-16 promotes the cytoplasmic localization of PQM-1 (Tepper et al., 2013). PQM-1 undergoes a progressive shift from nuclear to cytoplasmic localization as worms reach adulthood (Dowen et al., 2016) that continues during adult aging (Tepper et al., 2013). ChIP-seq data indicate that PQM-1 binds upstream of *sgk-1* (Tepper et al., 2013), suggesting that feedback mechanisms modulate the activity of the signaling pathway.

Dowen et al. (2016) found that when the function of *sgk-1* or hypodermal miRNA pathway components was lost, PQM-1 was inappropriately maintained in the nucleus during adulthood. These knockdowns also caused reduced intestinal fat stores, as determined by staining with the lipophilic dye Oil Red O, suggesting a wider disruption of lipid metabolism as a result of the perturbation of hypodermis to TORC2 signaling. It was later proposed that *pqm-1* functions in concert with *ceh-60* (see section “Other Regulators”), and possibly *unc-62* (see section “Tissue-, Stage- and Sex-Specific Regulation”; Dowen, 2019).

*sgk-1* was independently identified as an activator of *vit-2* in response to high concentrations of iron (Wang et al., 2016).

#### Regulation by SKN-1

*skn-1* encodes a transcription factor, distantly homologous to mammalian Nrf proteins. In addition to a role in embryonic development, *skn-1* coordinates systemic detoxification responses under normal conditions or in response to acute oxidative stress (An and Blackwell, 2003; Oliveira et al., 2009). *skn-1* may also play a broader role in coordinating lipid homeostasis (Steinbaugh et al., 2015). *skn-1* is known to be a downstream target of both the IIS and TOR pathways (Tullet et al., 2008; Robida-Stubbs et al., 2012). *skn-1* likely plays a role in regulating the vitellogenins, and vice versa.

**FIGURE 8:**
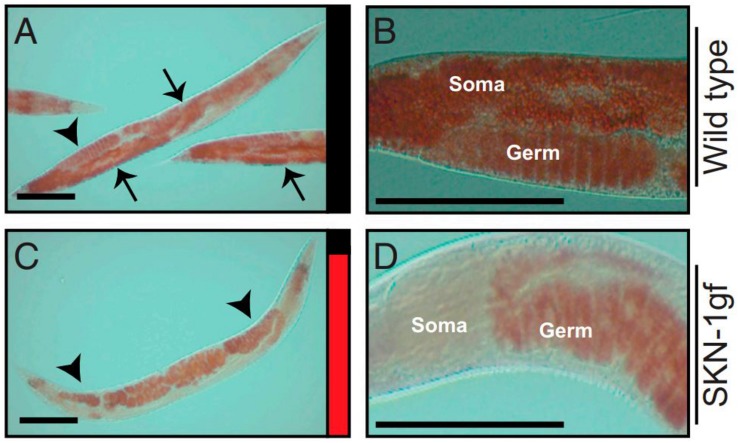
Age-dependent somatic depletion of fat in skn-1 gain of function mutants. Fixed post-reproductive worms are stained with Oil Red O, a lipid stain. Arrows indicate soma and arrowheads indicate germline. **(A,B)**, wildtype worms. **(C,D)**, skn-1 gain of function mutant. Scale bars, 100 μm. Reprinted from Lynn et al. (2015).

Lynn et al. (2015) described how, in *skn-1* gain-of-function mutants, lipid stores are transferred to the germline from the intestine rapidly after the cessation of reproduction, a phenomenon christened “age-dependent somatic depletion of fat” or ASDF (Figure 8). RNAi against vitellogenins prevents ASDF, suggesting that vitellogenins act as the mechanism by which *skn-1* causes intestinal depletion and germline accumulation of lipid stores. Specific lipids also regulate ASDF - dietary supplementation of the monounsaturated fatty acid (MUFA) oleic acid, lacking in *skn-1* gain-of-function mutants, could suppress ASDF.

In turn, vitellogenin accumulation in the worm may influence *skn-1* activity. The longevity phenotype of germline-less *glp-1* mutants requires *skn-1*. Steinbaugh et al. (2015) suggested that the pseudocoelomic accumulation of yolk in *glp-1* mutants caused by the removal of the yolk sink induces a stress response mediated by *skn-1*, resulting in the longevity phenotype. Indeed, knockdown of the yolk receptor *rme-2*, also causing yolk build up in the pseudocoelom, induced nuclear accumulation of SKN-1 and increased stress resistance, although studies disagree on the effect of *rme-2* RNAi on longevity (Seah et al., 2016; Dowen, 2019).

#### Other Regulators

*mafr-1* encodes a conserved corepressor of RNA pol III transcription. By its repression of tRNA and ribosomal RNA synthesis it affects the biosynthetic capacity of an organism - indeed in worms *mafr-1* RNAi increases body size, while overexpression reduces it (Khanna et al., 2014). *mafr-1* can act downstream of TORC1 signaling (Pradhan et al., 2017). *mafr-1* also regulates selected RNA pol II transcripts, among them the vitellogenins that are repressed by *mafr-1* (Khanna et al., 2014; Pradhan et al., 2017). *mafr-1* knockdown or overexpression altered the total lipid storage of worms. Mutations in either *daf-18* or *daf-16* abrogate the effect of *mafr-1* alteration on lipid stores, so *mafr-1* likely acts upstream of the IIS pathway to regulate lipid homeostasis. However, in a *daf-16* mutant background *mafr-1* RNAi still upregulated *vit-2*, *vit-4*, and *vit-5* but curiously not *vit-6*, suggesting differential regulation by IIS of the most diverged member of the vitellogenins (Khanna et al., 2014).

*kri-1* is a conserved gene with ankyrin repeats originally identified in reverse genetic screens for genes required for *daf-16* dependent lifespan extension in response to germline loss. In this capacity *kri-1* functions to promote DAF-16 nuclear localization in the intestine (Berman and Kenyon, 2006). Goszczynski et al. (2016) found that *kri-1* knockdown in the intestine strongly repressed the *vit-2* promoter and endogenous *vit-2* transcripts in a *daf-16* independent manner.

Goszczynski et al. (2016) also identified a forkhead-domain transcription factor, FKH-9, as a direct transcriptional activator for the 44 bp enhancer element of the *vit-2* promoter, with a binding site between the ELT-2 and MAB-3 binding sites. However, the effect of *fkh-9* RNAi on the full 2.7 kb promoter region was undetectable.

Van Rompay et al. (2015) identified *lrp-2* as a necessary activator of *vit-2* expression in a forward genetic screen. *lrp-2* is a member of the LDL receptor superfamily with a wide expression pattern, detected in body wall muscle, hypodermis and neurons. *lrp-2* has a largely flat expression profile during development, with a spike in expression evident around the L3-L4 molt. *lrp-2* mutants retain eggs, resulting in “bag-of-worms” phenotype in which internal hatching of larvae kills the mother, who is consumed from the inside by her larvae.

Van Rompay et al. (2015) also identified another regulator of vitellogenesis, *ceh-60*, which was later independently verified (Dowen et al., 2016; Dowen, 2019). *ceh-60* encodes a DNA binding homeobox protein which acts cell-autonomously in the intestine to regulate vitellogenesis and interacts directly with both UNC-62 (see section “Tissue-, Stage- and Sex-Specific Regulation”) and PQM-1 (see section “Target-of-Rapamycin (TOR) Signaling”). CEH-60 binds directly to the promoters of the vitellogenins (Dowen, 2019). Van Rompay et al. (2015) also identified a novel protein-encoding gene, *vrp-1* (vitellogenin regulating protein 1), which is expressed in intestinal nuclei and is regulated by, and in turn regulates, *ceh-60*. Both genes display expression spikes around the L3-L4 and L4-adult molts, and both mutants fail to activate vitellogenesis at the latter molt. *ceh-60* mutants have very strongly reduced survival of L1 starvation, likely mediated by lack of maternal vitellogenin production.

### Regulation by Extra-Intestinal Tissues

Many signaling pathways act systemically, with various components of the pathway, as constructed by classical genetics, actually operating within different tissues (Libina et al., 2003). Here we briefly highlight control of vitellogenin expression in the intestine by other tissues, presumably operating through signaling pathways already reviewed or as-yet undiscovered mechanisms.

#### Nervous System

As the proximal sites of environmental sensing, neurons are likely to be important in the fine-tuning of vitellogenesis by various pathways in response to environmental conditions. Indeed, the expression of the Sma/Mab TGF-β pathway ligand *dbl-1* and the insulin receptor homolog *daf-2* are largely neuronal (Li et al., 2014; Ramakrishnan et al., 2014). *dbl-1* has been shown to influence vitellogenin expression in a cell non-autonomous manner (Goszczynski et al., 2016).

Another regulator of vitellogenins, *ceh-60*, was proposed by Van Rompay et al. (2015) to act in neurons based on the apparent localization of a fluorescent reporter gene exclusively to the chemosensory neurons. However, *ceh-60* was later shown to be also expressed in the intestine and to regulate vitellogenesis cell-autonomously (Dowen, 2019; see section “Other Regulators”).

#### Hypodermis

The hypodermis is important for regulating the timing of developmental events in *C. elegans* - many so-called “heterochronic” mutants act in the hypodermis to control the timing of adult cell fate determination (Vella and Slack, 2005). The hypodermis is also important for the initiation of vitellogenesis in the intestine at the L4-adult molt.

In a forward genetic screen for regulators of the *vit-3* promoter, Dowen et al. (2016) found *alg-1*, an Argonaute protein that is an essential cofactor for miRNA biogenesis. miRNAs are short RNA molecules (around 22 nt in length) that bind to 3′ untranslated regions (UTRs) to negatively regulate their target mRNAs (Bartel, 2004). This led them to investigate the role of miRNAs in the initiation of vitellogenesis. *lin-4* and *let-7* are miRNAs that regulate the L1-L2 molt and L4-adult molt, respectively. While *let-7* has a broader expression pattern, *lin-4* expression is confined to neurons and the hypodermis. Loss-of-function mutants for both *lin-4* and *let-7* fail to initiate vitellogenesis at the L4-adult molt. Knockdown of the downstream mRNA targets negatively regulated by these miRNAs could partially rescue expression from the *vit-3* promoter.

*lin-29* encodes a zinc transcription factor normally expressed in the hypodermis during the L4 stage as the culmination of sequential miRNA expression during development. *lin-4* and *let-7* mutants do not express *lin-29* at the L4 stage. RNAi against *lin-29* in the hypodermis, but not the intestine, caused failure to initiate vitellogenesis at the L4-adult molt. Likewise, expression of *let-7* under a hypodermal promoter, but not an intestinal promoter, could rescue the vitellogenesis defect of a *let-7* null mutant. Surprisingly hypodermal or intestinal expression of *lin-29* could rescue a *lin-29* null mutant, implying that although *lin-29* is only expressed in the hypodermis, it acts through a regulator that can influence vitellogenesis in either a cell-autonomous or cell non-autonomous manner. The authors ultimately found that *lin-29* in the hypodermis activates the *sgk-1* kinase through TORC2 signaling in the intestine, as reviewed above.

#### Germline

The germline can exert a profound influence on somatic tissues, as demonstrated by the longevity of germline-less *glp-1* mutants. We have already seen that a component of the “germline pathway” required for this longevity, *kri-1*, acts in the intestine to promote vitellogenesis, although it is not clear whether it responds in this context to extra-intestinal signaling (Berman and Kenyon, 2006; Goszczynski et al., 2016). However, some instances of regulation of vitellogenesis by the germline have been described.

In a forward genetic screen for receptor-mediated endocytosis mutants that accumulate yolk at high levels in the pseudocoelom, Balklava et al. (2016) identified *pitr-1*, encoding an inorganic phosphate membrane transporter. Surprisingly, rather than disrupting yolk endocytosis, this mutant caused yolk build-up by increased vitellogenin expression at both the mRNA and protein levels. This effect was corroborated with a second null allele and systemic RNAi against *pitr-1*. *pitr-1* mutants also had a low brood size and partial penetrance of embryonic inviability.

Examining the expression pattern of *pitr-1*, the authors found the bulk of expression occurred in the germline at all developmental stages. Exclusive expression in the germline under a *pie-1* promoter could rescue the brood size defect and, incredibly, restored normal expression levels of vitellogenin, although embryonic viability was unaffected. This result suggests the existence of an uncharacterized pathway that requires this phosphate transporter in order to transduce signals from the germline to the intestine, thereby repressing vitellogenin expression.

DePina et al. (2011) also found that the germline can affect vitellogenin transcription. In *fem-1* mutants with a feminized germline, vitellogenin transcription was repressed, although protein levels appeared to be unaffected. *fem-3* mutants with a masculinized germline exhibited normal levels of transcription. This suggests the existence of a sperm-derived signal that promotes vitellogenin transcription.

### Environmental Regulation

#### Mating

DePina et al. (2011) found that mating increased transcription of vitellogenins and prevented the age-related decline of mRNA levels in a *daf-2* independent manner. Protein levels did not appear to be affected, although this may be a result of the extended reproductive span of outcrossed hermaphrodites, as suggested in section “Insulin/Insulin-Like Growth Factor Signaling (IIS),” for *daf-2* mutants. These results are consistent with the repression of vitellogenin transcription in the absence of sperm in *fem-1* mutants (see section “Germline”). However, as other physiological effects apparently caused by mating can be induced merely by male-conditioned media (Maures et al., 2014), this stimulation of vitellogenin transcription upon mating may be due to the presence of males rather than a sperm-derived signal, as the authors suggest.

Surprisingly, mating can affect vitellogenin expression in males. Shi et al. (2017) demonstrated that, as in hermaphrodites (Shi and Murphy, 2014), mating causes males to shrink and die. Both expression profiling by microarrays and a *vit-2:gfp* transgene indicated germline-dependent ectopic expression of vitellogenin in mated males. The reduced lifespan of mated males was found to be dependent on *unc-62* and *pqm-1*, both established regulators of vitellogenins (see sections “Tissue-, Stage- and Sex-Specific Regulation” and “Target-of-Rapamycin (TOR) Signaling,” respectively), although the effect of mutations in these genes on ectopic vitellogenin expression was not shown directly.

#### Ancestral Experience

Rechavi et al. (2014) found that ancestral experience of starvation can influence expression of vitellogenins. The authors starved L1 larvae for 6 days before recovery and sequenced small RNAs in these worms and in subsequent generations. Strikingly, differentially expressed heritable small RNAs aligned antisense to all 6 vitellogenin genes in the F3 progeny, indicating that an inter-generational memory of starvation influences vitellogenin expression. The authors suggested that double stranded RNA normally required for biogenesis of small RNAs could be derived from transcription of genes or non-coding RNAs lying on the opposite DNA strand near the 5′ or 3′ends of all of the vitellogenin coding sequences. Inheritance of differential small RNA expression required the genes *hrde-1* and *rde-4*. Despite the number of inherited small RNAs complementary to all of the vitellogenins, only *vit-4* appeared to be differentially expressed in the parental and F3 generations, suggesting that the effect of transgenerational memory of environmental experience on vitellogenin expression is modest.

#### Oxidative Stress

As reviewed in section “Regulation by SKN-1,” in *skn-1* gain-of-function mutants vitellogenins mediate the age-dependent depletion of somatic fat, or ASDF. In wildtype worms a full-scale ASDF response is induced within 12 h of exposure to hydrogen peroxide. Conversely, antioxidant treatment leads to accumulation of excess somatic fat (Lynn et al., 2015). These results suggest that *skn-1* regulates vitellogenin mobilization as part of a response to acute oxidative stress.

## Possible Alternative Functions of Vitellogenins in *C. elegans*

The presence of multiple vitellogenin paralogs in the *C. elegans* genome, and the substantial sequence divergence of *vit-6*, suggests the possibility of their co-option for other functions (Tufail and Takeda, 2008). Another indication that this may be the case is the expression of vitellogenins in males to a similar degree as in hermaphrodites at the L4 stage (Celniker et al., 2009). In the light of the various alternative functions known in other taxa (reviewed above), it is tempting to speculate on what other physiological or regulatory roles *C. elegans* vitellogenins may play. However, few papers have described any secondary functions for vitellogenins in worms.

As in the honeybee *A. mellifera* (Seehuus et al., 2006), vitellogenins may provide an antioxidant capacity in worms. Nakamura et al. (1999) found that *vit-6* was preferentially carbonylated in aging worms, suggesting a protective role. Sornda et al. (2019) found that *vit-6* protects against oxidative stress, although *vit-1 to -5* appeared to offer no protection. The idea of vitellogenins as antioxidants is also consistent with the dramatic mobilization of somatic fat in response to hydrogen peroxide, which may indicate secretion of vitellogenins as part of an acute oxidative stress response (Lynn et al., 2015). Support for this hypothesis from other studies is mixed (Inoue and Nishida, 2010; Fischer et al., 2013, 2014).

Only a single paper has suggested that vitellogenins act directly against pathogens in worms (Fischer et al., 2013). Since in adult hermaphrodites vitellogenins are found in pseudocoelomic circulation, vitellogenin function as an opsonin, as in fish (Liu et al., 2009), could facilitate phagocytosis of pathogens by the coelomocytes, scavenging cells anchored in the pseudocoelom. Additionally, vitellogenin uptake could hypothetically serve as an intergenerational immunity signal. Marré et al. (2016) found that exogenous dsRNA can be exported from the intestine and taken up along with yolk in an *rme-2* dependent manner, although this effect was not necessarily due to direct binding to vitellogenins as fluorescent dye injected into the pseudocoelom was also taken up. This non-specific uptake of pseudocoelomic contents could include pathogen-associated molecular patterns (PAMPs) or pathogen-derived nucleic acids in systemic circulation, thereby priming progeny innate immunity, as in *A. mellifera* (Salmela et al., 2015).

## Summary

Vitellogenins are yolk proteins found in almost all oviparous taxa. They transport a variety of lipids and micronutrients from adult tissues to oocytes. In addition, they can have other roles, in immune function, oxidative damage protection or social regulation. In *C. elegans*, 6 vitellogenin genes are very highly expressed in the intestine, although they do not appear to be strictly required for embryonic development. Rather, vitellogenin provisioning can impact on post-embryonic phenotypes and represent an intergenerational mechanism by which parental physiology can influence progeny outcomes. Indeed, the massive expression of *C. elegans* vitellogenins is regulated by various signaling pathways, such as the IIS, TOR, and TGF-β pathways, acting in the intestine or in extra-intestinal tissues, and can also be regulated by environmental experience. In *C. elegans*, alternative functions of vitellogenins, besides nutrient provisioning to progeny, are unclear and warrant further investigation.

## Author’s Note

Figures 1, 2, 3, and 6 originally appeared in MP’s doctoral thesis submitted to Pompeu Fabra University (UPF), Barcelona.

## Author Contributions

MP wrote the first draft of the text which was revised and edited by BL. Both authors listed have made a substantial, direct and intellectual contribution to the work, and approved it for publication.

## Conflict of Interest Statement

The authors declare that the research was conducted in the absence of any commercial or financial relationships that could be construed as a potential conflict of interest.
